# Variability in Tidal Volume Affects Lung and Cardiovascular Function Differentially in a Rat Model of Experimental Emphysema

**DOI:** 10.3389/fphys.2017.01071

**Published:** 2017-12-18

**Authors:** Caio G. R. S. Wierzchon, Gisele Padilha, Nazareth N. Rocha, Robert Huhle, Mariana S. Coelho, Cintia L. Santos, Raquel S. Santos, Cynthia S. Samary, Fernanda R. G. Silvino, Paolo Pelosi, Marcelo Gama de Abreu, Patricia R. M. Rocco, Pedro L. Silva

**Affiliations:** ^1^Laboratory of Pulmonary Investigation, Carlos Chagas Filho Biophysics Institute, Federal University of Rio de Janeiro, Rio de Janeiro, Brazil; ^2^Department of Anaesthesiology and Intensive Care Medicine, Pulmonary Engineering Group, University Hospital Carl Gustav Carus, Dresden University of Technology, Dresden, Germany; ^3^Department of Surgical Sciences and Integrated Diagnostics, Ospedale Policlinico San Martino, IRCCS for Oncology, University of Genoa, Genoa, Italy

**Keywords:** variable ventilation, respiratory system elastance, cardiorespiratory function, lung morphometry, inflammation, surfactant protein-D

## Abstract

In experimental elastase-induced emphysema, mechanical ventilation with variable tidal volumes (V_T_) set to 30% coefficient of variation (CV) may result in more homogenous ventilation distribution, but might also impair right heart function. We hypothesized that a different CV setting could improve both lung and cardiovascular function. Therefore, we investigated the effects of different levels of V_T_ variability on cardiorespiratory function, lung histology, and gene expression of biomarkers associated with inflammation, fibrogenesis, epithelial cell damage, and mechanical cell stress in this emphysema model. Wistar rats (*n* = 35) received repeated intratracheal instillation of porcine pancreatic elastase to induce emphysema. Seven animals were not ventilated and served as controls (NV). Twenty-eight animals were anesthetized and assigned to mechanical ventilation with a V_T_ CV of 0% (BASELINE). After data collection, animals (*n* = 7/group) were randomly allocated to V_T_ CVs of 0% (VV_0_); 15% (VV_15_); 22.5% (VV_22.5_); or 30% (VV_30_). In all groups, mean V_T_ was 6 mL/kg and positive end-expiratory pressure was 3 cmH_2_O. Respiratory system mechanics and cardiac function (by echocardiography) were assessed continuously for 2 h (END). Lung histology and molecular biology were measured post-mortem. VV_22.5_ and VV_30_ decreased respiratory system elastance, while VV_15_ had no effect. VV_0_, VV_15_, and VV_22.5_, but not VV_30_, increased pulmonary acceleration time to pulmonary ejection time ratio. VV_22.5_ decreased the central moment of the mean linear intercept (D2 of Lm) while increasing the homogeneity index (1/β) compared to NV (77 ± 8 μm vs. 152 ± 45 μm; 0.85 ± 0.06 vs. 0.66 ± 0.13, *p* < 0.05 for both). Compared to NV, VV_30_ was associated with higher interleukin-6 expression. Cytokine-induced neutrophil chemoattractant-1 expression was higher in all groups, except VV_22.5_, compared to NV. IL-1β expression was lower in VV_22.5_ and VV_30_ compared to VV_0_. IL-10 expression was higher in VV_22.5_ than NV. Club cell protein 16 expression was higher in VV_22.5_ than VV_0_. SP-D expression was higher in VV_30_ than NV, while SP-C was higher in VV_30_ and VV_22.5_ than VV_0._ In conclusion, VV_22.5_ improved respiratory system elastance and homogeneity of airspace enlargement, mitigated inflammation and epithelial cell damage, while avoiding impairment of right cardiac function in experimental elastase-induced emphysema.

## Introduction

The need for ventilator support in chronic obstructive pulmonary disease (COPD) is a result of respiratory failure (MacIntyre and Huang, 2008) due to a persistent chronic inflammatory response (GOLD, 2017). Noninvasive ventilation is widely used in COPD patients and is associated with shorter length of hospital stay, lower mortality rates, and lower costs as compared to invasive methods (Lindenauer et al., 2014). On the other hand, patients with more severe disease may still require invasive mechanical ventilation (Stefan et al., 2015a,b). In volume-controlled mechanical ventilation, due to its inherent monotonous pattern, the amplitude and duration of inflation and deflation are comparable. This, taking into account the time-constant inhomogeneity observed in COPD, can lead to delayed inflation of some lung areas and overdistension in others (Laghi et al., 2001; MacIntyre and Huang, 2008). This scenario may predispose to the development of ventilator-induced lung injury (VILI), further increasing the impedance of the pulmonary vascular bed and worsening the impact of mechanical ventilation on right ventricular (RV) function, which is already impaired in COPD (Vieillard-Baron et al., 1999; Wrobel et al., 2015).

Variable ventilation (VV), which is characterized by breath-to-breath variation of tidal volume (V_T_), has been shown to improve oxygenation (Lefevre et al., 1996) and respiratory function (Mutch et al., 2000; Spieth et al., 2009b; Thammanomai et al., 2013) reducing lung damage in models of acute lung injury (Kiss et al., 2016). Such beneficial effects have been ascribed to the potential of VV to recruit the lungs (Ruth Graham et al., 2011), redistribute pulmonary perfusion (Gama de Abreu et al., 2008), increase surfactant release (Arold et al., 2009), and even reduce the pro-inflammatory response of type-I alveolar epithelial cells (Rentzsch et al., 2017). Importantly, VV also improves distribution of ventilation across lung areas with different time constants (Huhle et al., 2016).

Recently, our group showed that variable tidal volumes set to a 30% coefficient of variation (CV) may result in a more homogenous distribution of ventilation, but might also impair right heart function in an elastase-induced emphysema model (Henriques et al., 2016). This setting of 30% CV was based on experiments performed in models of acute lung injury (Spieth et al., 2009b; Kiss et al., 2016). In theory, since emphysema and acute lung injury present distinct lung structural and functional changes, we hypothesized that a different setting of CV could improve both lung and cardiovascular function in experimental elastase-induced emphysema.

In the present study, we sought to determine the effects of different levels of CV on: (1) respiratory system elastance (E) and resistance (R); (2) gas exchange; (3) cardiac function, which was assessed by echocardiography measuring cross-sectional right and left ventricular diastolic areas (RV and LV, respectively), pulmonary acceleration time (PAT) to pulmonary ejection time (PET) ratio (PAT/PET) (an indirect index of pulmonary arterial hypertension), and left ventricular ejection fraction (EF) and fractional shortening (FS); and (4) gene expressions of biological markers associated with inflammation, epithelial cell damage, mechanical cell stress, and fibrogenesis in lung tissue.

## Materials and methods

### Ethics statement

This study was approved by the Ethics Committee of the Health Sciences Center (CEUA-CCS 059-15), Federal University of Rio de Janeiro. Animals received humane care in compliance with the Principles of Laboratory Animal Care formulated by the National Society for Medical Research and the U.S. National Academy of Sciences *Guide for the Care and Use of Laboratory Animals*.

### Animal preparation and experimental protocol

**Figure 1:**
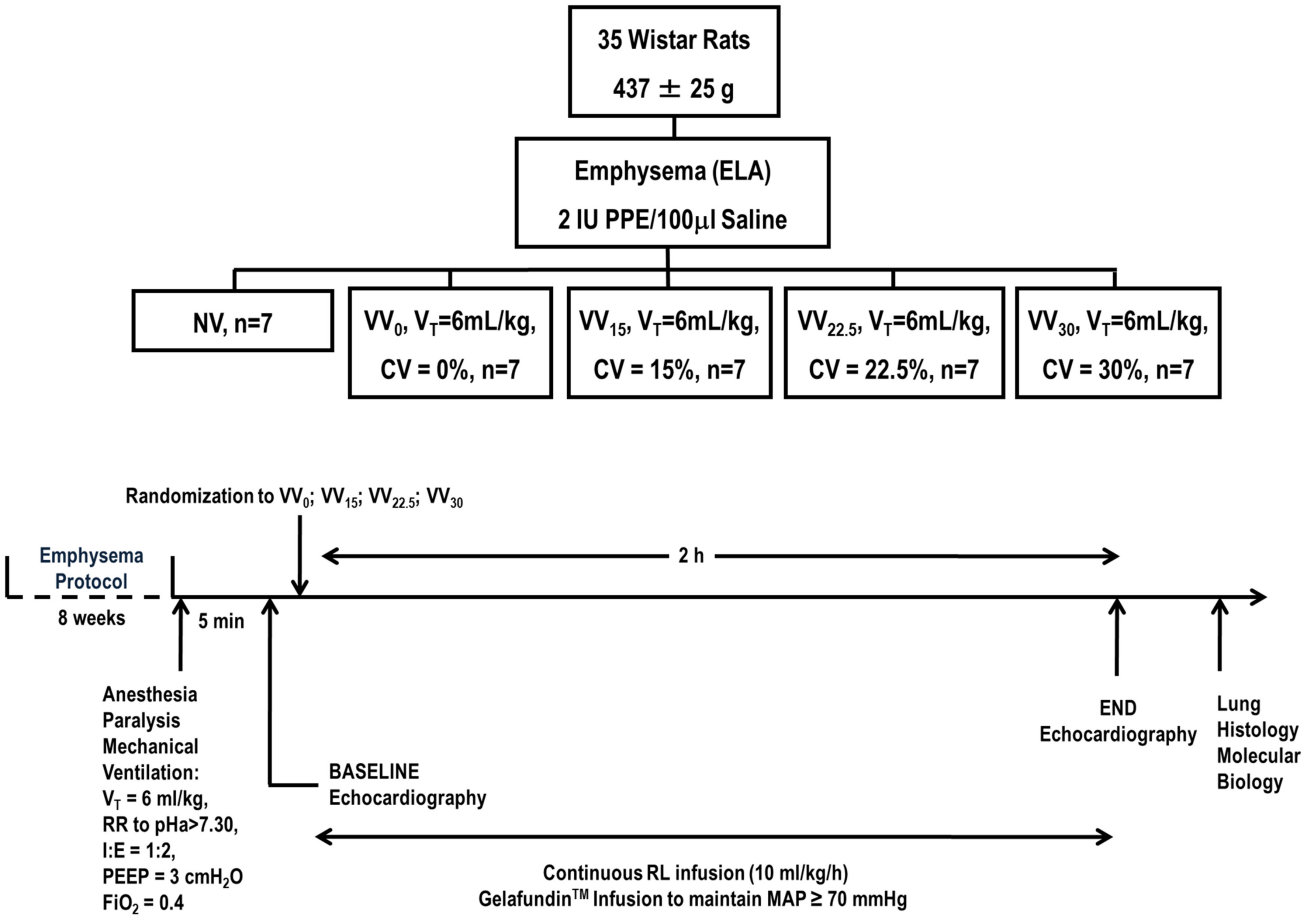
Design and timeline of experiments. PPE: porcine pancreatic elastase; NV: non-ventilated animals; VV: variable ventilation; V_T_: tidal volume; CV: coefficient of variation; RR: respiratory rate; I:E: inspiratory/expiratory ratio; PEEP: positive end-expiratory pressure; FiO_2_: fraction of inspired oxygen.

The time course of interventions is depicted in Figure 1. Emphysema was induced in 35 Wistar rats (weight 437 ± 25 g) according to a protocol described in detail elsewhere (Henriques et al., 2016). Briefly, animals received porcine pancreatic elastase (PPE) (2 IU in 0.1 mL of saline solution, Sigma Chemical Co., St. Louis, MO, USA) intratracheally, once weekly for 4 weeks. Before each intratracheal instillation, animals were premedicated with intraperitoneal diazepam (10 mg/kg, Compaz®, Cristália, Itapira, SP, Brazil) and anesthetized with 1.5–2.0% isoflurane (Cristália, SP, Brazil) by mask. Five weeks after the last instillation, experiments were performed.

Seven animals were non-ventilated (NV), serving as a control group. The remaining 28 animals were anesthetized with intraperitoneal diazepam (10 mg/kg, Compaz®, Cristália, Itapira, SP, Brazil), ketamine (50–100 mg/kg, Ketamin-S+®, Cristália, Itapira, SP, Brazil), and midazolam (2 mg/kg, Dormire®, Cristália, Itapira, SP, Brazil). An intravenous catheter (Jelco 24G) was inserted into the tail vein for continuous infusion of midazolam (2 mg/kg/h), ketamine (50 mg/kg/h), and Ringer's lactate (7 mL/kg/h, B. Braun, Crissier, Switzerland). Anesthetized animals were kept in the dorsal recumbent position and tracheotomized via a midline neck incision after subcutaneous injection of 2% lidocaine (Xylestesin®, Cristália, Itapira, SP, Brazil). The right internal carotid artery was cannulated (18G, Arrow International, USA) for blood sampling and mean arterial pressure (MAP) measurement. Heart rate (HR), MAP, and rectal temperature were continuously monitored (Networked Multiparameter Veterinary Monitor LifeWindow 6000V, Digicare Animal Health, Florida, USA). Body temperature was maintained at 37.5 ± 1°C using a heating bed. Gelafundin® 4% (B. Braun, São Gonçalo, RJ, Brazil) was administered intravenously in 0.5-mL boluses as needed to keep MAP ≥70 mmHg. Neuromuscular blockade was achieved by intravenous administration of pancuronium bromide (2 mg/kg, Pancuron®, Cristália, Itapira, SP, Brazil) and animals were mechanically ventilated (Inspira®, Harvard Apparatus, Holliston, Massachusetts, USA) in VCV mode with V_T_ = 6 mL/kg, respiratory rate (RR) adjusted to maintain arterial pH in the 7.35–7.45 range, inspired oxygen fraction (Fio_2_) = 0.4, and positive end-expiratory pressure (PEEP) = 3 cmH_2_O. After hemodynamic stabilization, respiratory system mechanics, arterial blood gases (Radiometer ABL80 FLEX, Copenhagen NV, Denmark), and echocardiographic parameters were measured (BASELINE). Following this step, animals were randomly assigned using the sealed-envelope method to one of four mechanical ventilation groups (*n* = 7/group), based on the CV of V_T_: (1) 0% (VV_0_); (2) 15% (VV_15_); (3) 22.5% (VV_22.5_); or (4) 30% (VV_30_). VV was applied on a breath-to-breath basis as a sequence of randomly generated V_T_ values (Gaussian distribution, *n* = 1200; mean V_T_ = 6 mL/kg), according to adjusted CV of V_T_ (nVentInspira) (Huhle et al., 2014), for 2 h. At the end of the experiment (END), echocardiographic parameters, respiratory system mechanics, and arterial blood gases were analyzed. Heparin (1000 IU) was injected intravenously; animals were killed by overdose of intravenous sodium thiopental (50 mg/kg, Thiopentax®, Cristália, Itapira, SP, Brazil) and their lungs extracted at PEEP = 3 cmH_2_O for lung morphometry and molecular biology analyses (Figure 1).

### Echocardiography

Shaved animals were placed in the dorsal recumbent position. Transthoracic echocardiography was performed by an expert (NNR) blinded to group allocation, using an UGEO HM70A system (Samsung, São Paulo, Brazil) equipped with a linear phased-array probe (8–13 MHz). Images were obtained from the subcostal and parasternal views. Short-axis two-dimensional views were acquired at the level of the papillary muscles to measure LV and RV areas. Left ventricular fractional shortening (FS) was calculated in M-mode, while the ejection fraction (EF) was obtained by multiplying LV outflow tract area by the volume time integral (VTI) on the LV long parasternal view. Pulsed-wave Doppler was used to measure PAT, PET, and the PAT/PET ratio. Heart ratio (HR) and the diameter of the inferior vena cava (IVC) and right atrium (RA) were assessed from the subcostal view. All parameters followed American Society of Echocardiography and European Association of Cardiovascular Imaging recommendations (Thibault et al., 2010; Lang et al., 2015).

### Data acquisition and respiratory system mechanics

Airflow, volume, and airway pressure were continuously recorded with a computer running custom software written in LabVIEW (National Instruments; Austin, Texas, USA) (Silva et al., 2013). All signals were amplified in a three-channel signal conditioner (TAM-D HSE Plugsys Transducers Amplifiers, Module Type 705/2, Harvard Apparatus, Holliston, Massachusetts, USA) and sampled at 1,000 Hz with a 12-bit analog-to-digital converter (National Instruments; Austin, Texas, USA). E and R were calculated offline based on the equation of motion (Uhlig et al., 2014).

### Lung morphometry

Morphometric analysis was performed in lungs excised at end-expiration (PEEP = 3 cmH_2_O). Immediately after removal, the left lung was flash-frozen by immersion in liquid nitrogen, fixed with Carnoy's solution, and paraffin-embedded. Sections (4 μm thick) were cut and stained with hematoxylin and eosin. An investigator (MSC) blinded to the origin of the material performed the microscopic examination. Morphometric analysis was done using an integrating eyepiece with a coherent system made of a 100-point grid, consisting of 50 lines of known length, coupled to a conventional light microscope (Axioplan, Zeiss, Oberkochen, Germany). Airspace enlargement was assessed by measuring the mean linear intercept (Lm) between alveolar walls at a magnification of × 400 (Hsia et al., 2010). To characterize the heterogeneity of airspace enlargement, the central moment of the mean linear intercept (D_2_ of Lm) was computed from 20 airspace measurements (Parameswaran et al., 2006), according to Equation 1:

(1) $$\begin{array}{l} {\text{D}}_{2}=\mu \cdot \left(1+\frac{\sigma^{2}}{\mu^{2}+\sigma^{2}}\right)\cdot \left(2+\sigma \cdot \frac{\gamma }{\mu }\right) \end{array}$$

where μ is the mean, σ the variance of airspace diameters, and γ the skewness of the diameter distribution. After D_2_ calculation, the homogeneity index (1/β) was derived from Lm and D_2_ values by their ratio.

### Molecular biology

Quantitative real-time reverse transcription polymerase chain reaction (qRT-PCR) was performed to assess the gene expression of interleukin (IL)-6, IL-1β, and IL-10, cytokine-induced neutrophil chemoattractant (CINC)-1, amphiregulin, surfactant protein (SP)-D and SP-C, club cell protein (CC)16, and type III procollagen (PCIII). Central slices of the right lung were cut, collected in cryotubes, flash-frozen by immersion in liquid nitrogen, and stored at −80°C. Total RNA was extracted from frozen tissues using the RNeasyPlus Mini Kit (Qiagen, Hilden, Germany) following the manufacturer's recommendations. RNA concentration was measured by spectrophotometry in a NanoDrop ND-1000 system (Thermo Scientific, Wilmington, DE, USA). First-strand cDNA was synthesized from total RNA using a QuantiTect reverse transcription kit (Qiagen, Hilden, Germany). The primers used are described in the online supplement (Table S1). Relative mRNA levels were measured by SYBR Green-based detection in an ABI 7500 Real-Time PCR system (Applied Biosystems, Foster City, California, USA). Samples were run in triplicate. For each sample, the expression of each gene was normalized to the acidic ribosomal phosphoprotein P0 (*36B4*) housekeeping gene and expressed as fold change relative to NV animals, using the 2^−ΔΔCt^ method, where ΔCt = Ct_target_ _gene_ − Ct_reference_ _gene_.

### Statistical analysis

Effect-size estimates were based on a previous study using the elastase instillation model of emphysema. A sample size of 7 animals per group would provide appropriate power (1–β = 0.8) to identify significant (α = 0.05) differences in E, taking into account an effect size d = 2.21, equal number of animals per group, a two-sided *t*-test, and multiple comparisons (*n* = 6) (α^*^ = 0.008, Bonferroni-adjusted) (G^*^Power 3.1.9.2, University of Düsseldorf, Germany).

Changes in variables between BASELINE and END were tested with a paired *t*-test. Two-way repeated-measures ANOVA followed by Holm-Šídák's multiple comparisons was used to compare cardiorespiratory function parameters among groups, while one-way ANOVA followed by Holm-Šídák's multiple comparisons was used to compare lung morphometry between NV and each CV group. Molecular biology variables were compared using the Kruskal–Wallis test followed by Dunn's multiple comparisons. Parametric data were expressed as mean ± standard deviation (SD) and non-parametric data as median (interquartile range). Associations of E with SP-D and SP-C were assessed using Spearman's correlation. All tests were performed in GraphPad Prism v6.07 (GraphPad Software, La Jolla, California, USA).

## Results

Respiratory and blood gas-exchange variables at BASELINE and END are reported in Table 1. V_T_, minute ventilation (${\text{V}}_{\text{E}}^{\prime }$), R, and PEEP levels did not differ among groups. As expected, the CV of V_T_ increased progressively across groups from VV_0_ to VV_30_. Compared to BASELINE, E was increased in VV_0_ and decreased in VV_22.5_, and VV_30_, which showed the lowest value at END. The arterial partial pressure of oxygen (PaO_2_) increased from BASELINE to END in all groups.

**Table 1:** Respiratory and blood gas exchange parameters at BASELINE and after 2 h (END).

		**VV_0_**	**VV_15_**	**VV_22.5_**	**VV_30_**
V_T_ (mL/kg)	BASELINE	6.0 ± 0.0	6.0 ± 0.0	6.0 ± 0.1	6.0 ± 0.1
	END	6.0 ± 0.1	6.0 ± 0.0	6.0 ± 0.1	6.1 ± 0.1
CV of V_T_ (%)	BASELINE	2.5 ± 1.9	1.2 ± 0.5	1.6 ± 0.7	1.4 ± 0.6
	END	1.6 ± 0.3	15.4 ± 0.9^*^#^‡^§	23.4 ± 1.3^*^#^†^§	28.8 ± 2.1^*^#^†^^‡^
${\text{V}}_{\text{E}}^{\prime }$ (mL/min)	BASELINE	149.5 ± 2.9	150.4 ± 1.9	149.8 ± 2.3	149.9 ± 2.3
	END	149.7 ± 2.1	153.1 ± 7.6	150.9 ± 3.6	151.8 ± 4.9
E (cmH_2_O/mL)	BASELINE	3.2 ± 0.5	3.3 ± 0.4	3.2 ± 0.6	3.0 ± 0.6
	END	3.6 ± 0.3^*^	3.2 ± 0.3	2.8 ± 0.2^*^	2.4 ± 0.2^*^#^†^
R (cmH_2_O/mL/s)	BASELINE	0.21 ± 0.04	0.20 ± 0.03	0.18 ± 0.02	0.19 ± 0.02
	END	0.18 ± 0.01	0.18 ± 0.04	0.16 ± 0.01	0.17 ± 0.02
PEEP (cmH_2_O)	BASELINE	3.2 ± 0.3	3.2 ± 0.2	3.2 ± 0.2	3.0 ± 0.4
	END	3.2 ± 0.3	3.2 ± 0.1	3.3 ± 0.3	3.1 ± 0.3
pHa	BASELINE	7.40 ± 0.02	7.38 ± 0.03	7.40 ± 0.05	7.41 ± 0.02
	END	7.35 ± 0.04	7.36 ± 0.04	7.36 ± 0.06	7.37 ± 0.07
PaO_2_ (mmHg)	BASELINE	139 ± 37	134 ± 38	133 ± 34	134 ± 37
	END	176 ± 21^*^	191 ± 18^*^	179 ± 26^*^	173 ± 20^*^
PaCO_2_ (mmHg)	BASELINE	37.7 ± 1.5	37.3 ± 3.1	38.4 ± 6.9	34.1 ± 3.0
	END	43.3 ± 5.3	36.6 ± 5.6	36.9 ± 8.0	38.3 ± 5.1
HCO_3_ (mmol/L)	BASELINE	23.6 ± 1.2	21.7 ± 1.6	23.4 ± 3.0	21.4 ± 2.3
	END	23.3 ± 3.4	20.4 ± 2.7	19.8 ± 2.7	21.8 ± 2.7

*Values are mean ± standard deviation (SD) of 7 emphysema animals in each group, exposed to elastase and analyzed 5 weeks after the last instillation. VV_0_: variable ventilation with 0% coefficient of variability in tidal volume; VV_15_: variable ventilation with 15% coefficient of variability in tidal volume; VV_22.5_: variable ventilation with 22.5% coefficient of variability in tidal volume; VV_30_: variable ventilation with 30% coefficient of variability in tidal volume; V_T_: tidal volume; CV of V_T_: coefficient of variation of tidal volume; V′E: minute ventilation; E: respiratory system elastance; R: respiratory system resistance; PEEP: positive end-expiratory pressure; pHa: arterial pH; PaCO_2_: arterial partial pressure of carbon dioxide; PaO_2_: arterial partial pressure of oxygen; HCO_3_: bicarbonate. Paired t-tests were used to compare BASELINE and END*.

* *vs. BASELINE (p < 0.05). Comparisons among ventilated groups were performed by two-way repeated-measures ANOVA followed by Holm-Šídák's multiple comparisons test*;

# *vs. VV_0_ (p < 0.05)*;

† *vs. VV_15_ (p < 0.05)*;

‡ *vs. VV_22.5_ (p < 0.05)*;

§ *vs. VV_30_ (p < 0.05)*.

**Figure 2:**
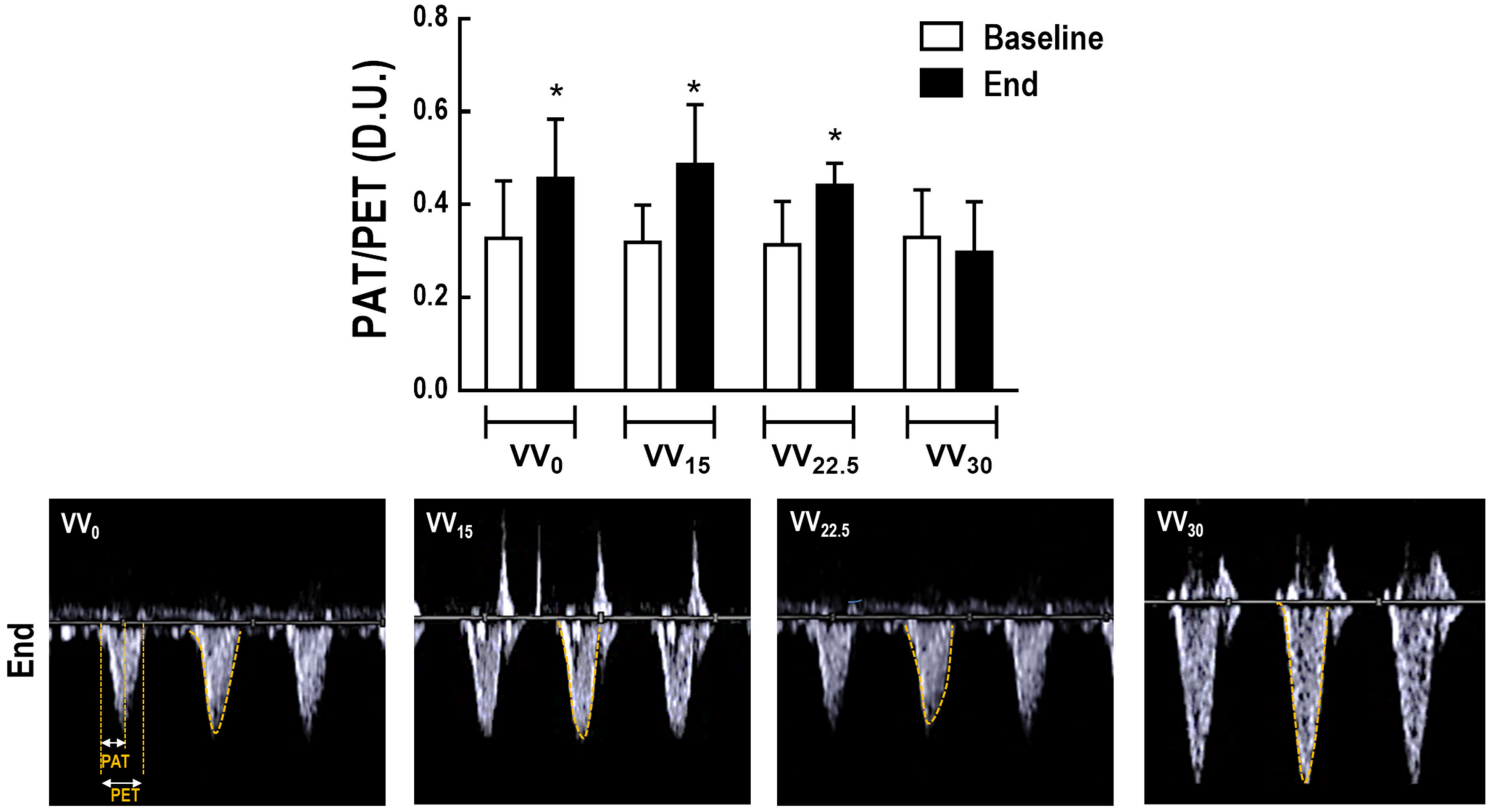
Cardiovascular function was assessed by echocardiography. Upper panel: PAT, pulmonary acceleration time; PET, pulmonary ejection time. Data presented as mean and standard deviation of 7 animals in all groups. The PAT/PET ratio was used as an indirect index of pulmonary arterial hypertension. (^*^) Significantly different from BASELINE (*p* < 0.05). Lower panel: representative images of pulmonary blood flow.

As depicted in Table 2, HR, MAP, cumulative fluids, IVC, RA, PET, LV area, EF, and FS did not differ among groups. RV area decreased from BASELINE to END in VV_0_, but increased in VV_30_. At END, VV_22.5_ and VV_30_ animals had larger RV area values than VV_0_; these were highest in VV_30_ compared to the other groups. Figure 2 shows the PAT/PET ratio and tracing of blood flow velocity measured at the pulmonary artery in representative animals. Compared to BASELINE, PAT/PET increased in all groups except VV_30_ at END, mainly due to increases in PAT, as shown in Table 2. Furthermore, VV_30_ animals had lower PAT/PET values compared to VV_0_ and VV_15_ animals.

**Table 2:** Hemodynamics, cumulative fluids, and echocardiography data.

		**VV_0_**	**VV_15_**	**VV_22.5_**	**VV_30_**
HR (bpm)	BASELINE	367 ± 75	351 ± 60	411 ± 66	348 ± 46
	END	351 ± 67	357 ± 52	371 ± 60	319 ± 89
MAP (mmHg)	BASELINE	116 ± 43	132 ± 17	133 ± 39	115 ± 26
	END	129 ± 17	105 ± 24	106 ± 36	118 ± 22
Cumulative fluids (mL)	BASELINE	–	–	–	–
	END	6.7 ± 1.9	7.5 ± 1.5	9.0 ± 2.2	6.5 ± 1.0
IVC diameter (cm)	BASELINE	0.19 ± 0.07	0.23 ± 0.09	0.14 ± 0.03	0.20 ± 0.06
	END	0.20 ± 0.10	0.21 ± 0.08	0.12 ± 0.03	0.21 ± 0.11
RA diameter (cm)	BASELINE	0.38 ± 0.05	0.43 ± 0.04	0.36 ± 0.05	0.40 ± 0.07
	END	0.36 ± 0.05	0.40 ± 0.03	0.30 ± 0.03	0.39 ± 0.05
RV area (cm^2^)	BASELINE	0.34 ± 0.06	0.38 ± 0.07	0.34 ± 0.07	0.37 ± 0.07
	END	0.28 ± 0.05^*^	0.39 ± 0.11	0.33 ± 0.05^#^	0.46 ± 0.12^*^#^‡^
PAT (ms)	BASELINE	20.7 ± 8.6	22.3 ± 7.5	19.2 ± 5.4	22.6 ± 7.8
	END	28.9 ± 9.8^*^	33.3 ± 12.1^*^	25.2 ± 5.5^*^	17.3 ± 8.3
PET (ms)	BASELINE	65.1 ± 18.9	69.0 ± 14.5	62.0 ± 8.5	68.8 ± 9.6
	END	65.0 ± 16.7	61.6 ± 13.4	56.9 ± 9.3	58.1 ± 13.6
LV area (cm^2^)	BASELINE	0.14 ± 0.04	0.12 ± 0.03	0.16 ± 0.08	0.13 ± 0.04
	END	0.20 ± 0.05	0.13 ± 0.04	0.19 ± 0.10	0.16 ± 0.08
EF (%)	BASELINE	95.2 ± 3.9	95.1 ± 2.3	95.7 ± 3.4	93.2 ± 6.6
	END	92.1 ± 5.4	89.5 ± 7.3	95.3 ± 1.9	87.9 ± 9.2
FS (%)	BASELINE	67.8 ± 9.2	66.4 ± 6.3	68.7 ± 9.2	64.4 ± 13.2
	END	61.7 ± 12.3	54.4 ± 12.8	66.1 ± 5.2	55.9 ± 14.3

Values are mean ± standard deviation (SD) of 7 animals in each group, exposed to elastase and analyzed 5 weeks after the last instillation. HR: heart rate; MAP: Mean arterial pressure; IVC: inferior vena cava; RA: right atrium RV: right ventricle; PAT: pulmonary acceleration time; PET: pulmonary ejection time; LV: left ventricle; EF: ejection fraction; FS: fractional shortening. Paired t-tests were used to compare BASELINE and END;

* *vs. BASELINE (p < 0.05). Comparisons among ventilated groups were performed by two-way repeated-measures ANOVA followed by Holm-Šídák's multiple comparisons test*;

# *vs. VV_0_ (p < 0.05)*;

*^†^ vs. VV_15_ (p < 0.05)*;

‡ *vs. VV_22.5_ (p < 0.05)*;

*§ vs. VV_30_ (p < 0.05)*.

**Figure 3:**
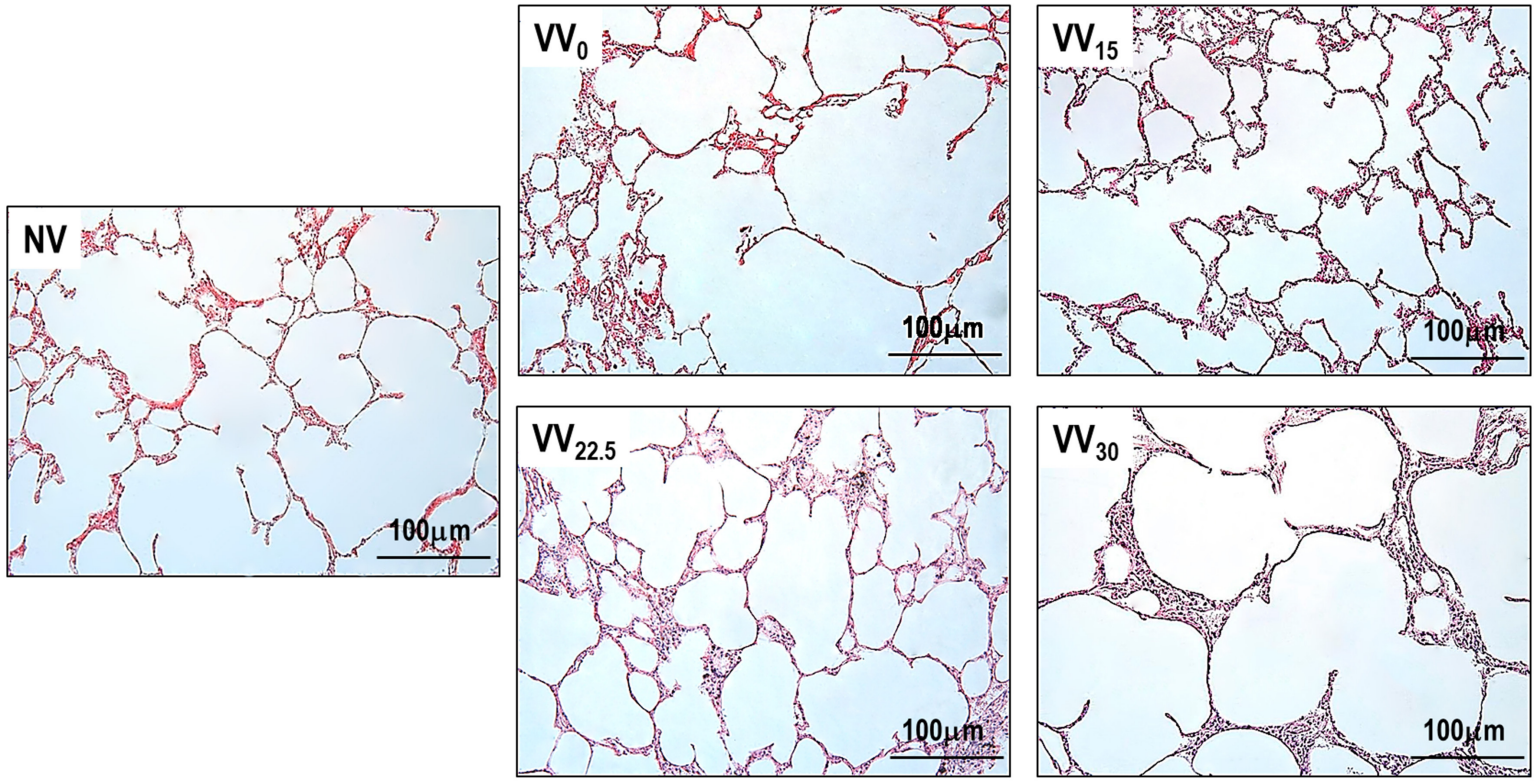
Representative light microscopy images. Original magnification ×200.

Figure 3 depicts light microscopy images of one representative animal per group. Morphometric data are shown in Table 3. VV_22.5_ presented the lowest values of mean linear intercept (Lm) among all groups, as well as a lower D_2_ of Lm compared to NV. Accordingly, the homogeneity index 1/β was higher in VV_22.5_ than in NV.

**Table 3:** Lung morphometry in mechanically ventilated animals.

	**NV**	**VV_0_**	**VV_15_**	**VV_22.5_**	**VV_30_**
Lm (μm)	96 ± 10	89 ± 13	89 ± 13	70 ± 6^*^#^†^§	88 ± 15
D_2_ of Lm (μm)	152 ± 45	131 ± 39	120 ± 45	82 ± 9^*^	117 ± 27
1/β	0.66 ± 0.13	0.70 ± 0.13	0.78 ± 0.14	0.85 ± 0.06^*^	0.76 ± 0.10

*Values are mean ± standard deviation (SD) of 7 animals in each group, exposed to elastase and analyzed 5 weeks after the last instillation. NV: non-ventilated; VV_0_: variable ventilation with 0% coefficient of variability in tidal volume; VV_15_: variable ventilation with 15% coefficient of variability in tidal volume; VV_22.5_: variable ventilation with 22.5% coefficient of variability in tidal volume; VV_30_: variable ventilation with 30% coefficient of variability in tidal volume; Lm: mean linear intercept; D_2_: central moments of mean linear intercept; 1/β: homogeneity index. Comparisons were performed by one-way analysis of variance followed by Holm-Šídák's multiple comparisons test*.

* *vs. NV (p < 0.05)*;

# *vs. VV_0_ (p < 0.05)*;

† *vs. VV_15_ (p < 0.05)*;

§ *vs. VV_30_ (p < 0.05)*.

**Figure 4:**
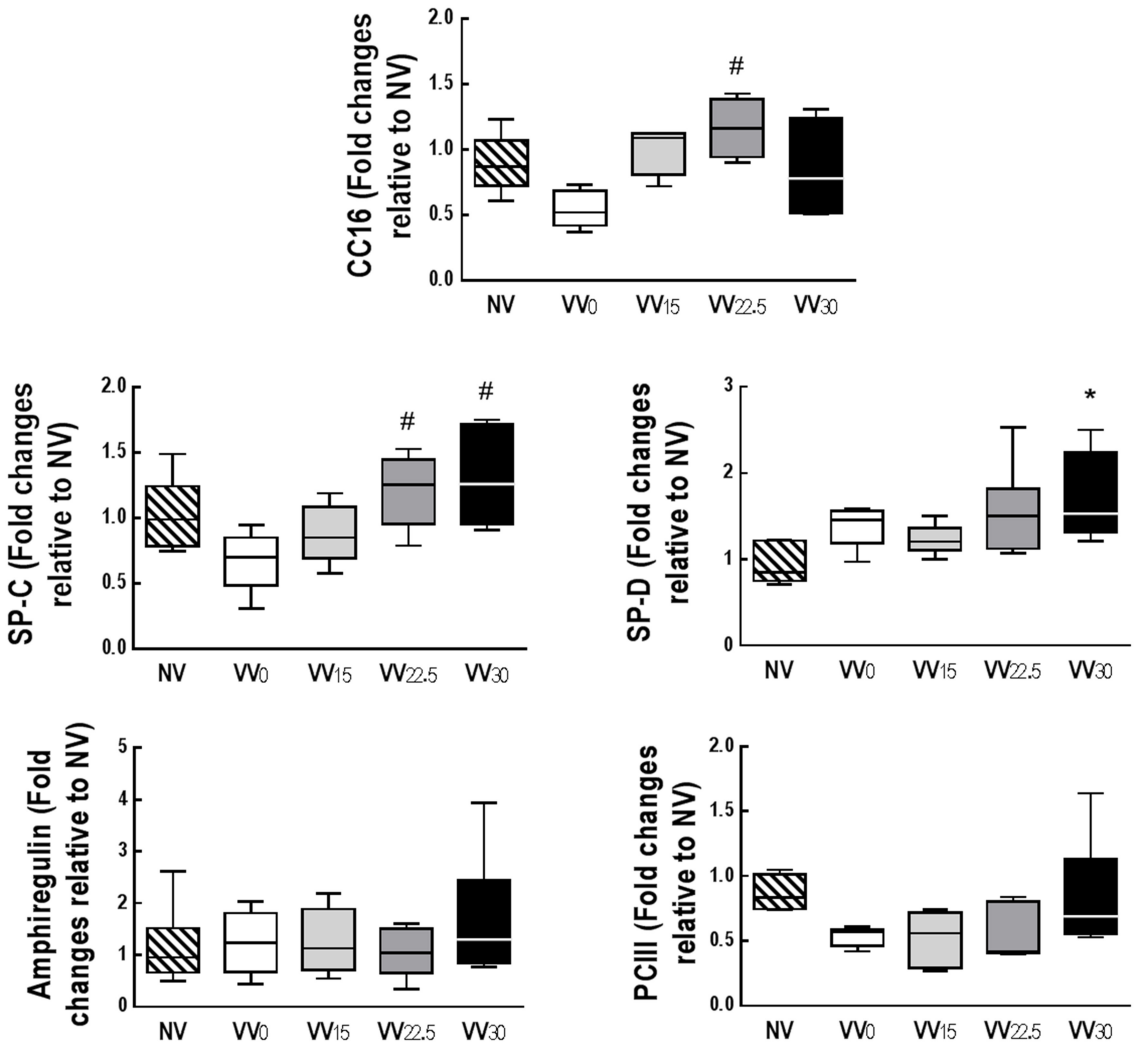
Quantitative real-time polymerase chain reaction analysis of biological markers of club cell protein (CC)-16, alveolar epithelial cells [surfactant proteins (SP)-C, SP-D], mechanical cell stress (amphiregulin), and fibrogenesis [type III procollagen (PCIII)]. Data are presented as a box plot. Lines denote the median and boxes delimit the 25th and 75th percentiles of 7 animals per group. Relative gene expression was calculated as a ratio of the average gene expression levels compared with the reference gene (*36B4*) and expressed as fold change relative to NV. ^*^Significantly different from NV group (*p* < 0.05); ^#^Significantly different from VV_0_ group (*p* < 0.05).

**Figure 5:**
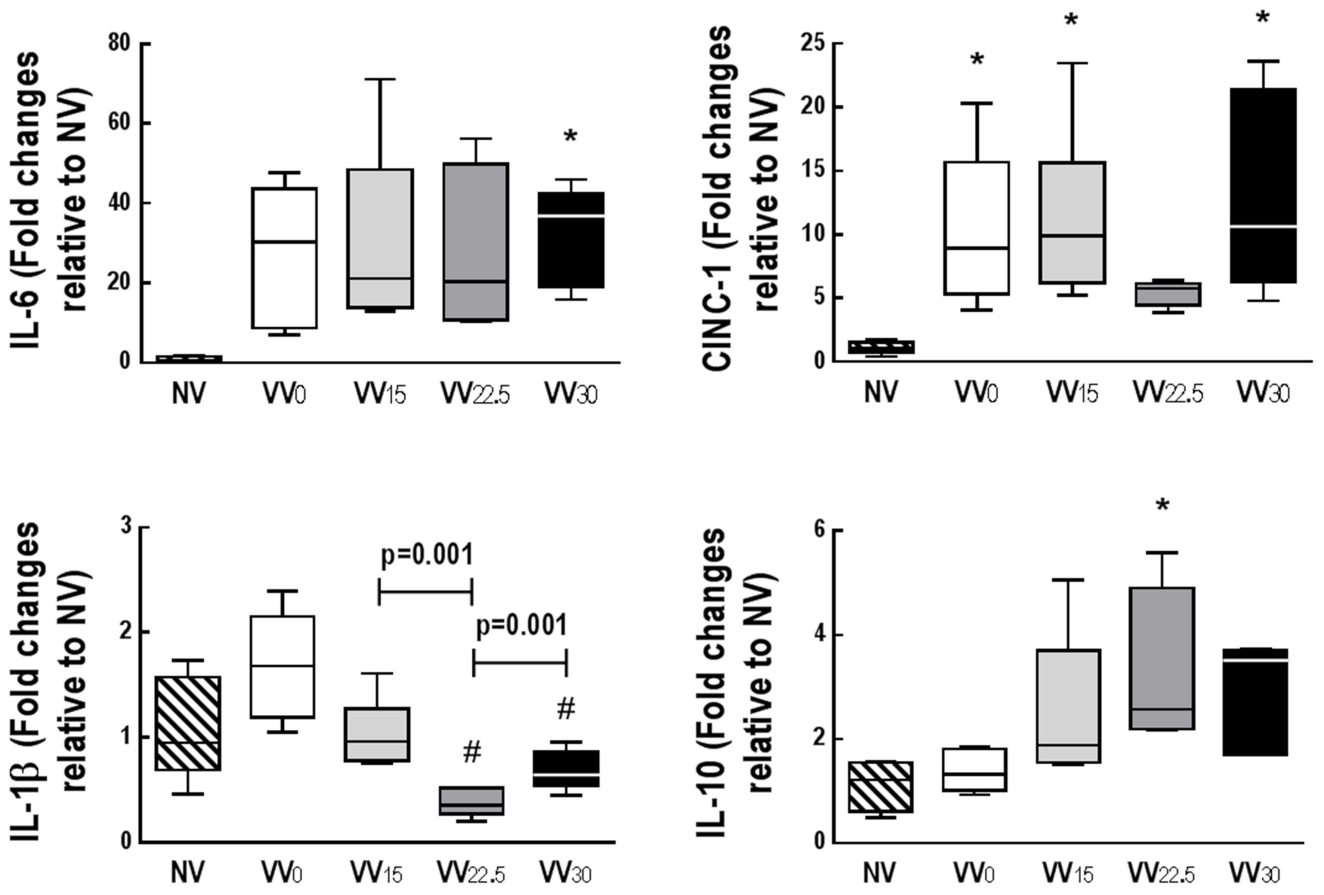
Quantitative real-time polymerase chain reaction analysis of biological markers of inflammation: interleukin (IL)-6, IL-1β, IL-10, and cytokine-induced neutrophil chemoattractant (CINC)-1. Data are presented as a box plot. Lines denote the median and boxes delimit the 25th and 75th percentiles of 7 animals per group. Relative gene expression was calculated as a ratio of the average gene expression levels compared with the reference gene (*36B4*) and expressed as fold change relative to NV. ^*^Significantly different from NV group (*p* < 0.05). #Significantly different from VV_0_ group (*p* < 0.05).

Gene expressions of amphiregulin and PCIII did not differ among groups. CC16 expression was higher in VV_22.5_ than VV_0_. Gene expression of SP-D was higher in VV_30_ than NV, while SP-C expression was higher in VV_30_ and VV_22.5_ than VV_0_ (Figure 4). Compared to NV, VV_30_ showed higher IL-6 expression. CINC-1 expression was higher in all groups except VV_22.5_ when compared to NV. IL-1β expression was lower in VV_22.5_ and VV_30_ compared to VV_0_. IL-10 mRNA expression was higher in VV_22.5_ than NV (Figure 5).

E correlated negatively with gene expressions of SP-D (*r* = −0.55, *p* = 0.007) and SP-C (*r* = −0.62, *p* = 0.002).

Table 4 depicted the positive and negative percentage alterations of the mean values in each group at END compared to BASELINE. Progressive improvement in E is noticeable, whereas PAT (the main contributor to PAT/PET ratio) deteriorated, suggesting pulmonary arterial hypertension. PaO_2_ improved in all groups compared to BASELINE.

**Table 4:** Mean percent changes for several parameters during VV_0_, VV_15_, VV_22.5_, and VV_30_ compared to BASELINE.

	**%VV_0_**	**%VV_15_**	**%VV_22.5_**	**%VV_30_**
E (cmH_2_O/mL)	+15.4	−3.8	−10.5	−17.6
PAT (ms)	+50.0	+46.2	+35.4	−20.7
PET (ms)	+4.9	−7.2	−7.0	−15.3
PAT/PET	+50.3	+58.0	+73.6	−4.4
RV area (cm^2^)	−17.9	+1.5	−2.2	+25.5
PaO_2_ (mmHg)	+32.7	+50.9	+42.2	+43.3

*Positive and negative mean percent changes in several parameters in each group compared to BASELINE (n = 7 emphysema animals). VV_0_: variable ventilation with 0% coefficient of variability in tidal volume; VV_15_: variable ventilation with 15% coefficient of variability in tidal volume; VV_22.5_: variable ventilation with 22.5% coefficient of variability in tidal volume; VV_30_: variable ventilation with 30% coefficient of variability in tidal volume; E: respiratory system elastance; PAT: pulmonary acceleration time; PET: pulmonary ejection time; RV: right ventricle; PaO_2_: arterial partial pressure of oxygen. Only statistically significant findings are presented*.

## Discussion

The main findings of the present study were that, in a rat model of experimental emphysema: VV_22.5_ and VV_30_ decreased E, while VV_15_ did not affect it; VV, regardless of CV, did not improve gas exchange; VV_0_, VV_15_, and VV_22.5_, but not VV_30_, increased PAT/PET; VV_22.5_ was associated with the lowest Lm, while increasing the homogeneity index compared to NV; CINC-1 and IL-1β expressions were lower in VV_22.5_ compared to NV and VV_0_, respectively; CC16 and IL-10 expressions were higher in VV_22.5_ compared to VV_0_ and NV, respectively; and VV_30_ led to higher expressions of SP-C and SP-D than VV_0_ and NV, respectively.

To the best of our knowledge, this was the first investigation of the effects of different levels of V_T_ variability on pulmonary and cardiovascular function, lung morphometry, and gene expression of markers of inflammation, surfactant proteins, epithelial cell damage, cell mechanical stress, and fibrogenesis in experimental emphysema. We chose the repeated intratracheal elastase instillation model because it reproduces important features of emphysema, including deterioration of respiratory system mechanics, airspace enlargement, and lung inflammation (Antunes and Rocco, 2011; Cruz et al., 2012; Henriques et al., 2016; Oliveira et al., 2016; Padilha et al., 2016; Rocha et al., 2017; Suki et al., 2017). Furthermore, multiple elastase instillations can lead to cardiorespiratory alterations (Antunes et al., 2014) that are consistent with *cor pulmonale*, including increased RV afterload (Henriques et al., 2016). We chose to conduct analyses 5 weeks after the last elastase instillation because this time point provides an adequate combination of cardiorespiratory function impairment and structural lung damage (Henriques et al., 2016). The levels of V_T_ variability were selected on the basis of our previous experience with variable ventilation in models of acute lung injury (Spieth et al., 2009b; Kiss et al., 2016); these studies showed that the optimal CV is situated in the range of 15 to 30% of V_T_.

Our finding that VV improved E is in agreement with studies in models of acute lung injury (Thammanomai et al., 2008; Spieth et al., 2009a; de Magalhaes et al., 2016; Kiss et al., 2016; Samary et al., 2016) and emphysema (Henriques et al., 2016). VV is able to recruit the lungs more efficiently than conventional recruitment maneuvers (Thammanomai et al., 2008; Spieth et al., 2009b), leading to an increase in aerated lung tissue (Ruth Graham et al., 2011) with consequent improvement of viscoelastic properties. In this line, Thammanomai et al. (2008) showed that, in injured mice, variable ventilation, but not conventional ventilation with periods of large breaths, resulted in less lung inflammation. In emphysema, a substantial degree of small airway narrowing can be present (McDonough et al., 2011), but deep inspirations, which are present sporadically during VV, have been shown to revert this (Wong et al., 2012). In addition, the fact that VV applies subphysiological V_T_ values may have helped limit hyperinflation during mechanical ventilation and promote emptying of lung regions with lower time constants. Although we did not observe differences in overall resistance, we cannot exclude that regional resistance might have been affected by tissue destruction (Hantos et al., 2008), as detected by better homogeneity index in lung morphometry. It is worth noting that elastance improved continuously with increasing variability in V_T_, and was lowest at VV_30_. Curiously, this behavior is similar to that described during VV in experimental acute lung injury (Spieth et al., 2009a), and the lack of a “U-shape” is likely explained by the fact that the highest variability was limited to a CV of 30%, which corresponds to a peak V_T_ around 10–12 mL/kg. Up to this V_T_ level, we were likely still located on the linear portion of the respiratory system pressure–volume curve (Ito et al., 2005). Our data also suggest that the improvement in elastance might be related to an increase in surfactant production. Release of surfactant is known to be models (Bartolak-Suki et al., 2017) and variable stretch of alveolar epithelial cells (Arold et al., 2009). Although we did not evaluate the cellular mechanisms of surfactant production in detail, mechanical stretch has been shown to stimulate surfactant production through epidermal growth factor receptor (EGFR) phosphorylation (Sanchez-Esteban et al., 2004). In addition, one possible explanation for the increase in surfactant mRNA synthesis would be improvement in overall cell bioenergetics. In this context, variable stretch has been shown to improve ATP production by promoting mitochondrial biogenesis, which, in turn, led to structural changes such as increased organization of the actin, microtubule, and mitochondrial networks, as characterized by their fractal dimension and coefficient of variation (Bartolak-Suki et al., 2015). By promoting cytoskeleton organization, the alveolar epithelial cells are more prone to start or continue surfactant synthesis into the alveolar space (Singh et al., 2004).

Interestingly, oxygenation increased in all groups but was not further improved by VV, suggesting that a time-dependent recruitment effect occurred. This is likely explained by the use of PEEP in all groups. Furthermore, we cannot exclude the possibility that redistribution of perfusion without significant degrees of recruitment was present. In fact, VV has been shown to improve oxygenation even when the fraction of non-aerated lung tissue increases (Gama de Abreu et al., 2008).

In agreement with a previous study from our group (Henriques et al., 2016), VV_30_ did not improve PAT/PET, but increased RV area. Taken together, the findings of these studies indicate that higher V_T_ variability impairs right heart function. The present study, however, adds to the present state of knowledge. Intermediate levels of variability, namely VV_15_ and VV_22.5_, improved PAT/PET to a similar extent as VV_0_, limiting impact on right ventricular afterload. One possible explanation for this behavior is that both excessive and insufficient lung-unit recruitment, which have been observed at extremes of V_T_ variability, led to high or low lung volumes respectively, thus increasing pulmonary vascular resistance (Simmons, 1961). This hypothesis is supported by our observation that an intermediate-to-high level of variability, namely VV_22.5_, resulted in a more homogeneous and less pronounced airspace enlargement, as suggested by lower Lm and D_2_ and higher homogeneity index (Parameswaran et al., 2006). In addition to opening the lungs and keeping them homogeneously opened, VV_22.5_ may better distribute mechanical forces, thus mitigating ventilator-induced injury. Even though IL-6 expression did not differ between VV0 and VV_22.5_, expression of other pro-inflammatory mediators, such as CINC-1 and IL-1β, was reduced in VV_22.5_, whereas gene expression of anti-inflammatory IL-10 increased. Differences in the behaviors of these pro-inflammatory mediators may be associated with the pathophysiology of emphysema and with differential cell activation during VILI.

Interestingly, despite improvement in respiratory system mechanics, gene expression of IL-6 was increased at VV_30_. This finding is in agreement with a previous study from our group showing that conventional and variable mechanical ventilation with a CV of 30% resulted in increased lung inflammation in experimental emphysema (Henriques et al., 2016). In this context, a recent study reported a significant reduction in IL-6 gene expression after 4 h of variable cyclic stretch in LPS-stimulated alveolar cells (similar to 30% of CV of V_T_ *in vivo*) as compared to monotonous cyclic stretch (Rentzsch et al., 2017). These effects were potentially mediated by the ERK1/2 pathway after mechanical stress. Activation of the ERK1/2 pathway by cell stretch reflects prior activation of TNFR-associated factors (TRAFs), mainly TRAF2 (Sotoudeh et al., 2002), which is critical to IKK activation. Once TRAFs are activated, this may elicit activation of NF-κB and AP-1 (Oeckinghaus et al., 2011), resulting in increased inflammatory gene expression. Although we did not measure ERK1/2 and TRAF, we speculate that, even taking into account the fragile tissue observed in emphysema (Suki et al., 2013), VV_22.5_ might not trigger this inflammatory pathway.

Importantly, variable ventilation did not result in increased cell mechanical stress or fibrogenesis, as indicated by amphiregulin and PCIII gene expressions, respectively. Nevertheless, VV_22.5_ positively modulated CC16 expression. CC16 is synthesized predominantly in the lungs, but also found in the circulation. Serum CC16 has been reported to decrease with lung disease progression (Vestbo et al., 2011) and smoking, and is considered a marker of bronchial cell dysfunction (Park et al., 2013). In this line, CC16 has been shown to play a protective role in COPD (Laucho-Contreras et al., 2015), and its levels should be maintained to protect the lungs from progression of COPD-like disease (Park et al., 2013). In the present study, VV_22.5_ resulted in higher CC16 expression compared to VV0.

### Possible implications for further studies

Our data suggest that, in patients with emphysema who require invasive mechanical ventilation, variation in tidal volumes may contribute to improved E and reduce the inhomogeneity of airspace enlargement, especially if a V_T_ CV of 22.5% is used. This variability level can also reduce the likely negative impact of variable ventilation on right heart function without increasing the pro-inflammatory and pro-fibrotic lung response. Clinical studies are necessary to determine the potential role of variable ventilation within this CV range in emphysema.

## Limitations

This study has several limitations. First, the emphysema model used herein (repeated intratracheal instillation of elastase) does not entirely reproduce the clinical picture seen in humans, and cannot be directly extended to other models of emphysema. Second, the mechanical ventilation period (2 h) was short. Long-term variable ventilation may lead to different results on analysis of inflammatory cell infiltration in lung tissue. Third, since we tested only four levels of variability, we cannot exclude the possibility that intermediate levels might lead to different results. Fourth, echocardiography was not gated by respiratory cycles, which may have affected measurement of cardiac function parameters. Nevertheless, the possibility of bias was minimized by the 15-min imaging periods. Fifth, although there are several forms of tidal volume distribution, we chose a Gaussian distribution for technical reasons, local settings, and experience (de Magalhaes et al., 2016; Henriques et al., 2016; Kiss et al., 2016; Soluri-Martins et al., 2017). For experts in the field of lung disease, it would be interesting to compare different distributions in order to extract the best readout in terms of cardiorespiratory interaction among them. Sixty, gene expression of biomarkers does not necessarily translate to increased protein levels; however, the relatively short period of intervention precluded protein analysis.

## Conclusion

In conclusion, VV_22.5_ improved respiratory system elastance and homogeneity of airspace enlargement, mitigated inflammation and epithelial cell damage, while avoiding impairment of right cardiac function in experimental elastase-induced emphysema.

## Author contributions

Conceived and designed the experiments: CW, GP, NR, RH, PP, MG, PR, and PS; Performed experiment: CW, GP, NR, MC, RS, CSS, and FS; Analyzed data: CW, GP, NR, MC, CLS, and RH; Interpreted results of research: CW, GP, NR, RH, PP, MG, PR, and PS; Drafted, edited, critically revised paper: CW, RH, PP, MG, PR, PS; All authors approved final version of manuscript.

### Conflict of interest statement

MG has been granted patents on variable pressure support ventilation. The other authors declare that the research was conducted in the absence of any commercial or financial relationships that could be construed as a potential conflict of interest.
