# Supplementary material for: Variability in Tidal Volume Affects Lung and Cardiovascular Function Differentially in a Rat Model of Experimental Emphysema
Source: Front Physiol. 2017 Dec 18;8:1071. doi: 10.3389/fphys.2017.01071 (PMC5741669; doi:10.3389/fphys.2017.01071)
Supplement: Supplementary file 1 [file Table1.docx]

**Supplemental Digital Content**

**SDC**

**Variability in Tidal Volume Differently Affects Lung and Cardiovascular Function in an Experimental Model of Emphysema in Rats**

Caio GRS Wierzchon^1^, Gisele A Padilha^1^, Nazareth N Rocha^1^, Robert Huhle^2^, Mariana S Coelho^1^, Cintia L Santos^1^, Raquel S Santos^1^, Cynthia S Samary^1^, Fernanda RG Silvino^1^, Paolo Pelosi^3##^, Marcelo Gama de Abreu^2##^, Patricia RM Rocco^1##^, Pedro L Silva^1##^

^1^Laboratory of Pulmonary Investigation, Carlos Chagas Filho Biophysics Institute, Federal University of Rio de Janeiro, Rio de Janeiro, Brazil.

^2^Department of Anaesthesiology and Intensive Care Medicine, Pulmonary Engineering Group, University Hospital Carl Gustav Carus, Dresden University of Technology, Dresden, Germany.

^3^Department of Surgical Sciences and Integrated Diagnostics, San Martino Policlinico Hospital, IRCCS for Oncology, University of Genoa, Genoa, Italy.

^##^Dr. Pelosi, Abreu, Rocco and Silva share senior authorship

**ADDITIONAL METHODS**

Table S1. Forward and reverse oligonucleotide sequences of target gene primers

| **Gene** | **Primer** | **Primer sequences (5′-3′)** |
| --- | --- | --- |
| CINC-1 | Forward | TGC ACC CAA ACC GAA GTC AT |
|  | Reverse | TTG TCA GAA GCC AGC GTT CAC |
| IL-6 | Forward | CTC CGC AAG AGA CTT CCA G |
|  | Reverse | CTC CTC TCC GGA CTT GTG A |
| IL-1β | Forward | CTA TGT CTT GCC CGT GGA G |
|  | Reverse | CAT CAT CCC ACG AGT CAC A |
| PCIII | Forward | ACC TGG ACC ACA AGG ACA C |
|  | Reverse | TGG ACC CAT TTC ACC TTT C |
| Amphiregulin | Forward | TTT CGC TGG CGC TCT CA |
|  | Reverse | TTC CAA CCC AGC TGC ATA ATG |
| SP-D | Forward | AAA TCT TCA GGG CGG CAA A |
|  | Reverse | GGC CTG CCT GCA CAT CTC |
| SP-C | Forward | CTGGTTACCACTGCCACCTT |
|  | Reverse | TCA AGA CTG GGG ATG CTC TC |
| CC16 | Forward | GAT CG CCA TCA CAA TCA CTG |
|  | Reverse | GGT ATC CAC CAG CCT CTT CA |
| IL10 | Forward | TCC CTG GGT GAG AAG CTG |
|  | Reverse | GCT CCA CTG CCT TGC TCT |
| 36B4 | Forward | AAT CCT GAG CGA TGT GCA G |
|  | Reverse | GCT GCC ATT GTC AAA CAC |

Primers used in experiments. CINC-1, cytokine-induced neutrophil chemoattractant 1; IL-6, interleukin-6; IL-1β, interleukin-1β; PCIII, pro-collagen III; SP-D and SP-C surfactant proteins D and C; CC16, club cell protein; IL-10, interleukin-10; 36B4, acidic ribosomal phosphoprotein P0.
